# Supplementary material for: Drug Discovery in Low Data Regimes: Leveraging a Computational Pipeline for the Discovery of Novel SARS-CoV-2 Nsp14-MTase Inhibitors
Source: bioRxiv. 2023 Nov 13:2023.10.03.560722. Originally published 2023 Oct 4. Preprint. [Version 2] doi: 10.1101/2023.10.03.560722 (PMC10592886; doi:10.1101/2023.10.03.560722)
Supplement: Supplementary file 1 [file NIHPP2023.10.03.560722v2-supplement-1.pdf]

bioRxiv preprint doi: https://doi.org/10.1101/2023.10.03.560722; this version posted November 13, 2023. The copyright holder for this preprint (which was not certified by peer review) is the author/funder, who has granted bioRxiv a license to display the preprint in perpetuity. It is made available under aCC-BY 4.0 International license.

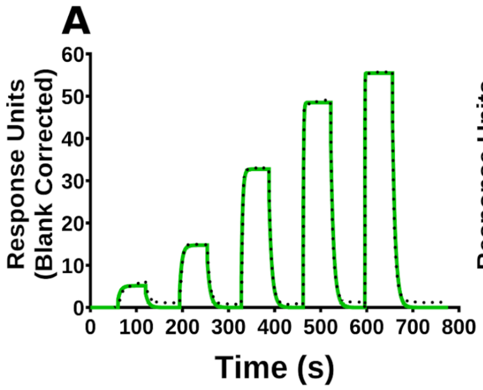

FIG. S1. **Orthogonal confirmation of NSC620333 binding to nsp14 by SPR. (A)** A representative sensorgram (solid green) is shown with the kinetic fit (black dots). From kinetic fitting, a K_D value of $427 \pm 84\, nM$, $k_{\rm on}$ of $3.2 \pm 0.15 \times 10^5\, M^{-1} S^{-1}$ and $k_{\rm off}$ of $1.3 \pm 0.2 \times 10^{-1}\, S^{-1}$ were determined. **(B)** The steady-state response (black circles) obtained from (A) is shown with the steady-state 1:1 binding model fitting (red dashed line). A steady-state K_D value of $544 \pm 22\, nM$ ($n = 3$) was also calculated. All values are presented as mean ± standard deviation of three independent experiments ($n = 3$).

Supplementary Information:

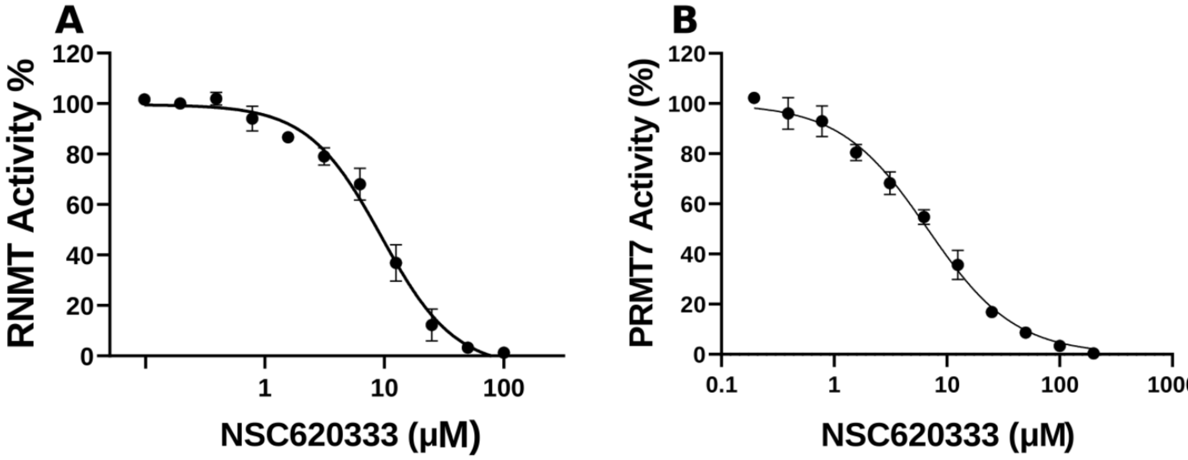

FIG. S2. **Inhibition of RNMT and PRMT7 activity by NSC620333.** (A) The IC_{50} value was determined for NSC620333 to be 8.6 ± 1.3 µM, Hill Slope: -1.5. (B) The IC_{50} value was determined for NSC620333 to be 7.0 ± 0.6 µM, Hill Slope: -1.9.

bioRxiv preprint doi: https://doi.org/10.1101/2023.10.03.560722; this version posted November 13, 2023. The copyright holder for this preprint (which was not certified by peer review) is the author/funder, who has granted bioRxiv a license to display the preprint in perpetuity. It is made available under aCC-BY 4.0 International license.

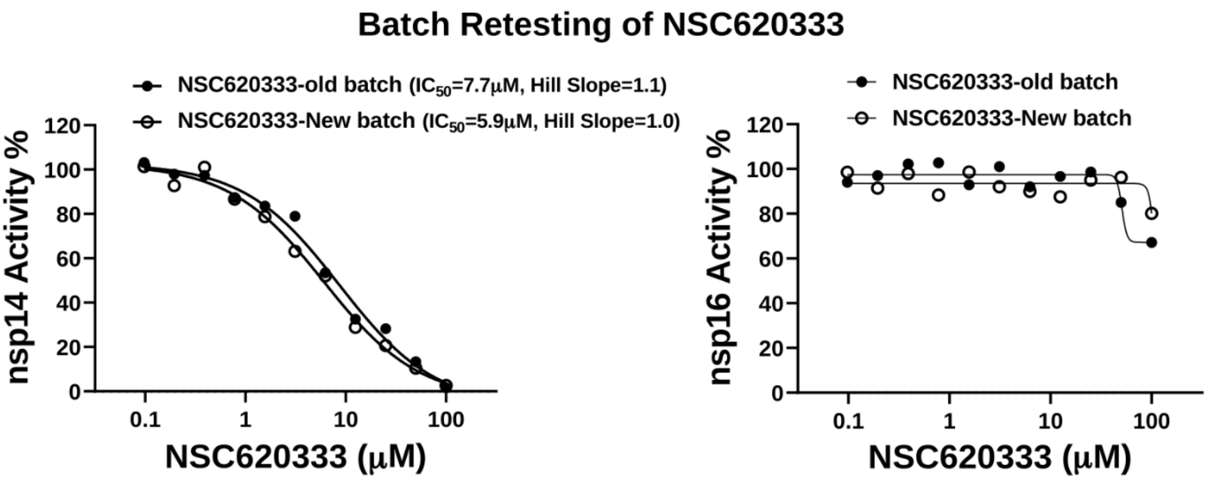

FIG. S3. **Inhibition Results from Batch Retesting of Purified NSC620333.** Outcomes of testing a custom-ordered, highly purified (>99%) sample of NSC620333. The exclusion of impurities culminated in a more pronounced inhibition of nsp14, underscoring the potency of the purified compound in the regulation of this complex.

bioRxiv preprint doi: https://doi.org/10.1101/2023.10.03.560722; this version posted November 13, 2023. The copyright holder for this preprint (which was not certified by peer review) is the author/funder, who has granted bioRxiv a license to display the preprint in perpetuity. It is made available under aCC-BY 4.0 International license.

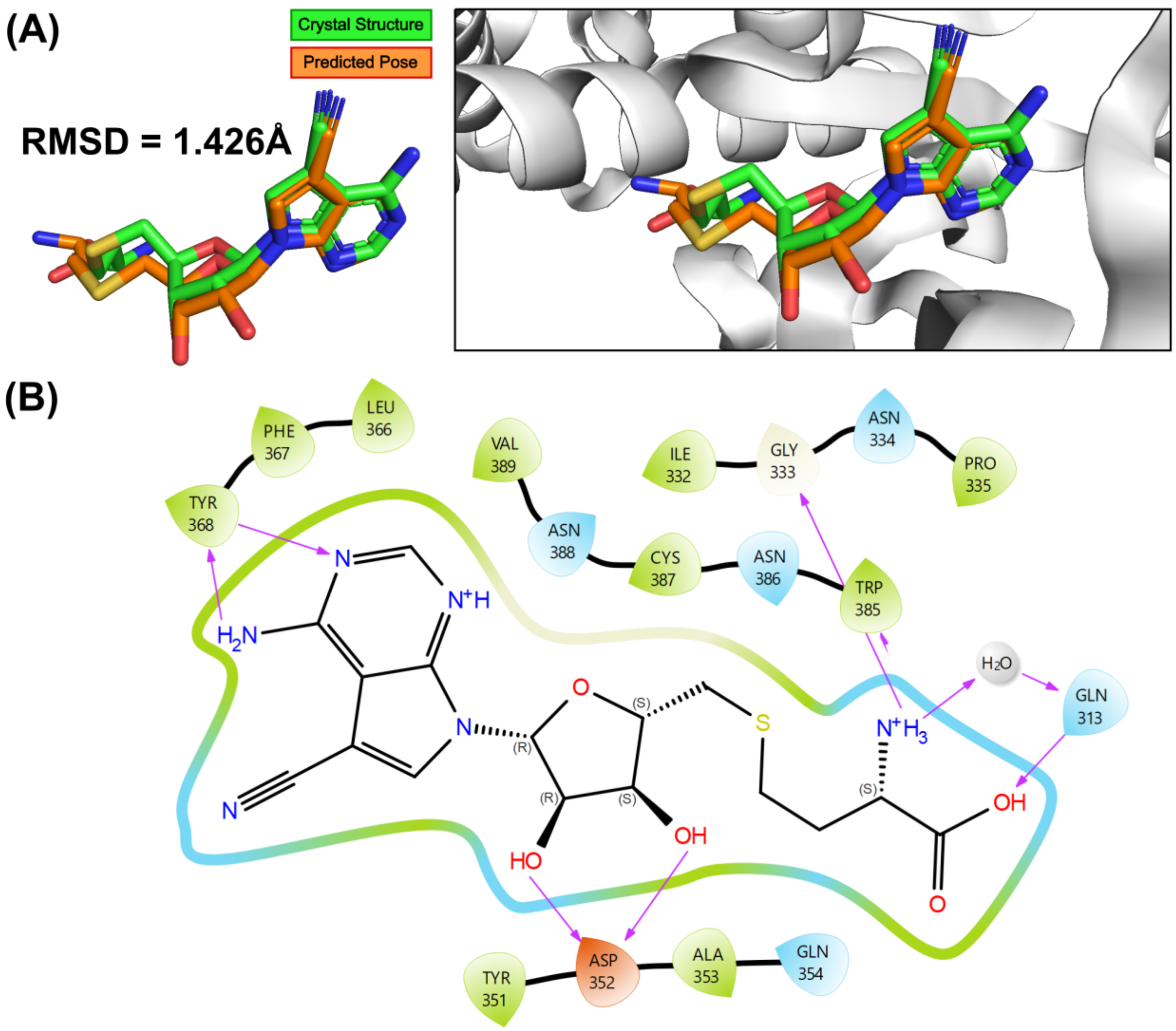

FIG. S4. **Comparative Analysis of the Crystal Structure and Predicted Docking Pose of SS148 in Complex with nsp14.** **(A)** Superimposition of SS148's actual crystal structure (depicted in green) and its predicted docking pose (depicted in orange), highlighting the high overlap between prediction and experiment. A Root Mean Square Deviation (RMSD) of 1.426Å is observed. **(B)** Two-Dimensional Interaction Diagram depicting the complex formation of SS148 with the nsp14 Methyltransferase (MTase), as per the actual crystal structure.

bioRxiv preprint doi: https://doi.org/10.1101/2023.10.03.560722; this version posted November 13, 2023. The copyright holder for this preprint (which was not certified by peer review) is the author/funder, who has granted bioRxiv a license to display the preprint in perpetuity. It is made available under aCC-BY 4.0 International license.

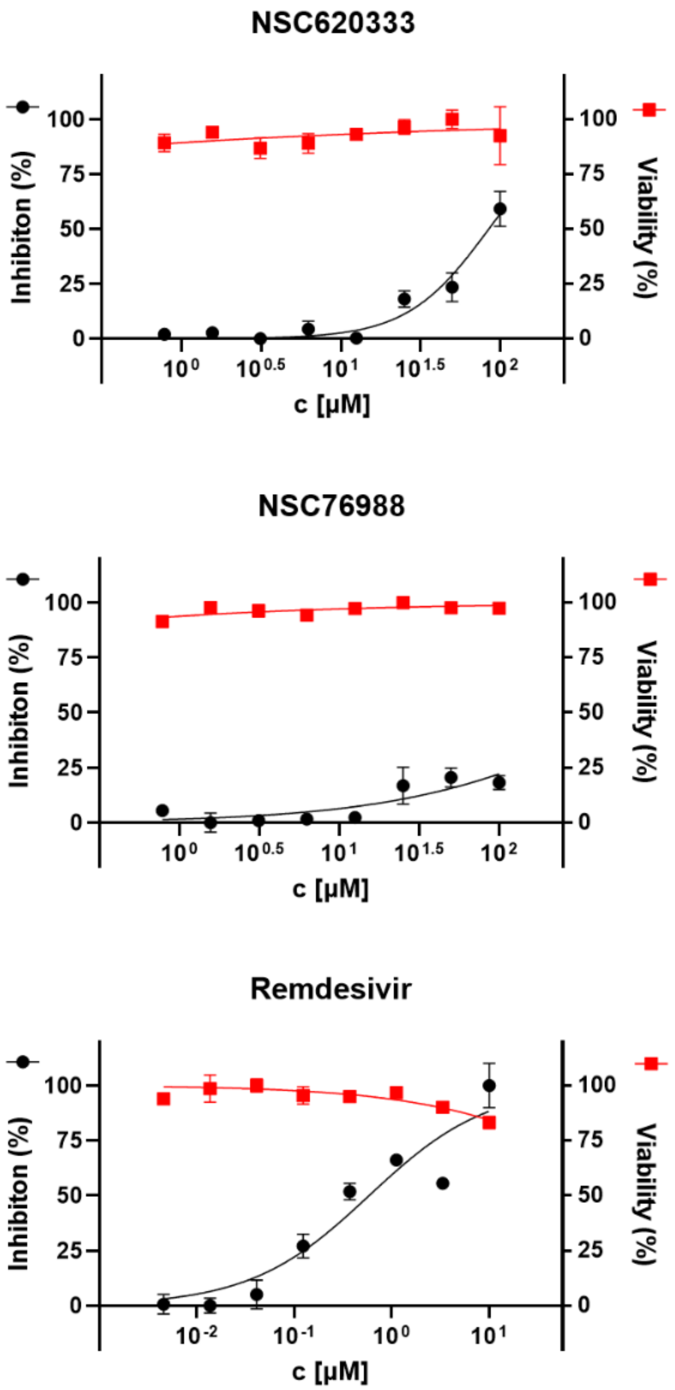

FIG. S5. **Anti-SARS-CoV-2 activity and cytotoxicity of NSC620333 and NSC76988 in Calu-3 cells.** Dose-response curve analysis of anti-SARS-CoV-2 activity (black circle) and cytotoxicity (red square) of NSC620333 and NSC76988 in Calu-3 cells. Remdesivir served as a control. All values are presented as mean ± standard deviations from experiments performed in triplicate.

bioRxiv preprint doi: https://doi.org/10.1101/2023.10.03.560722; this version posted November 13, 2023. The copyright holder for this preprint (which was not certified by peer review) is the author/funder, who has granted bioRxiv a license to display the preprint in perpetuity. It is made available under aCC-BY 4.0 International license.

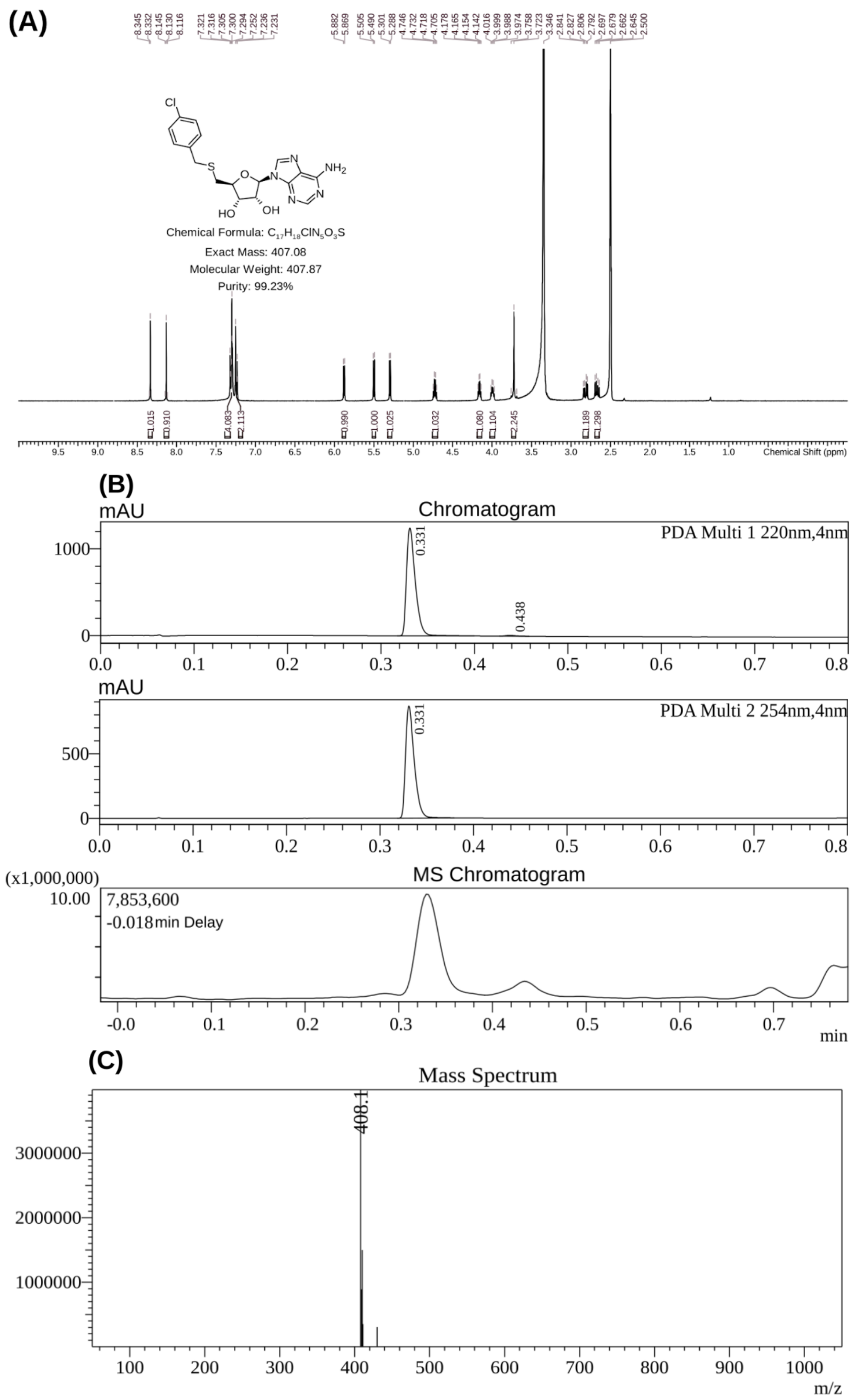

FIG. S6. Characterization data for chlorobenzothiophene 3. A. H-NMR spectrum. B. HPLC traces with both UV and MS detectors. C. High-resolution mass spectrum.

bioRxiv preprint doi: https://doi.org/10.1101/2023.10.03.560722; this version posted November 13, 2023. The copyright holder for this preprint (which was not certified by peer review) is the author/funder, who has granted bioRxiv a license to display the preprint in perpetuity. It is made available under aCC-BY 4.0 International license.

TABLE S2. **Binding Affinity predictions of lead compounds.** Predicted binding affinities of 40 lead compounds, in complex with nsp14, using 3 methods, is provided. In increasing order of accuracy, the methods are: Glide SP, Glide XP (docking based) and MM/GBSA. MM/GBSA results are provided without the entropic term, leading to an overestimation of the binding free energies.

Compound	Docking Score; Glide SP (kcal/mol)	Docking Score; Glide XP (kcal/mol)	MM/GBSA Score (kcal/mol)
NSC76988	-5.790	-6.184	-32.3499
NSC620333	-10.111	-10.728	-60.5078
NSC77131	-3.994	-3.330	-34.1961
NSC400718	-3.641	-5.061	-48.4538
NSC34443	-5.482	-7.552	-31.4914
NSC630814	-5.497	-6.385	-41.5536
NSC102798	-5.189	-5.831	-26.9979
NSC4348	-4.748	-6.385	-34.6630
NSC400937	-3.950	-3.729	-21.4589
NSC4624	-3.435	-3.437	-26.7526
NSC2269	-5.132	-5.203	-42.4074
NSC255523	-5.991	-7.907	-10.4218
NSC670682	-4.876	-5.666	-30.7842
NSC99790	-5.577	-9.373	-33.8143
NSC646375	-7.611	-7.247	-36.1022
NSC27605	-5.449	-5.275	-17.6311
NSC107661	-8.464	-9.472	-34.1128
NSC293892	-5.916	-6.851	-35.7854
NSC44037	-6.045	-5.975	-48.2280
NSC77680	-6.518	-7.769	-10.0810
NSC80136	-6.327	-5.874	-44.7396
NSC114010	-6.586	-8.365	-37.5531
NSC268226	-6.958	-8.510	-48.1415
NSC163444	-8.800	-9.520	-49.6504
NSC377438	-5.958	-6.242	-24.6196
NSC131119	-6.246	-7.424	-56.3134
NSC39302	-5.237	-6.365	-32.5813
NSC317609	-4.419	-5.521	-36.9720
NSC232469	-5.091	-5.590	-31.5946
NSC655184	-5.412	-5.619	-41.1487
NSC92432	-6.003	-7.781	-35.6097
NSC137050	-3.900	-5.217	-39.4464
NSC158437	-5.666	-4.750	-36.8949
NSC613624	-6.917	-8.477	-31.3669
NSC60360	-5.951	-7.539	-31.6140
NSC330685	-6.129	-6.713	-11.8248
NSC54251	-9.143	-10.080	-44.8260
NSC313453	-7.559	-7.261	-34.6341
NSC132916	-5.313	-6.259	-30.8526

bioRxiv preprint doi: https://doi.org/10.1101/2023.10.03.560722; this version posted November 13, 2023. The copyright holder for this preprint (which was not certified by peer review) is the author/funder, who has granted bioRxiv a license to display the preprint in perpetuity. It is made available under aCC-BY 4.0 International license.

TABLE S3. **Inhibitory Activity of NSC620333 on a Diverse Panel of 33 Human Methyltransferases (MTases).** Percent inhibitory activity of NSC62033 at a concentration of 10 µM across three replicates for each of the 33 human RNA-, DNA-, and protein-MTases. The average inhibitory activity and standard deviation for each MTase are also provided.

MTases	Activity (%) at 10 µM			
	Replicate 1	Replicate 2	Replicate 3	Average
G9a	97	79	91	89 ± 9
GLP	95	105	101	100 ± 5
SUV39H1	99	91	97	96 ± 4
SUV39H2	108	96	106	103 ± 7
SUV420H1	84	97	98	93 ± 8
SUV420H2	96	104	86	96 ± 9
PRMT1	97	94	84	91 ± 6
PRMT3	75	78	85	79 ± 5
PRMT4	96	95	101	97 ± 3
PRMT5	69	64	55	63 ± 7
PRMT6	97	99	103	100 ± 3
PRMT7	35	29	32	32 ± 3
PRMT8	97	99	93	96 ± 3
PRMT9	118	118	113	117 ± 3
PRDM9	110	105	104	106 ± 3
SETDB1	104	106	100	103 ± 3
SETD2	99	103	107	103 ± 4
SETD7	85	88	87	87 ± 1
SETD8	79	90	86	85 ± 6
SMYD2	104	114	99	105 ± 8
SMYD3	101	108	91	100 ± 9
MLL1	84	87	84	85 ± 2
MLL3	44	57	53	51 ± 6
EZH2	101	90	98	96 ± 5
BCDIN3D	57	66	64	62 ± 5
DOT1L	103	90	86	93 ± 9
ASH1L	113	97	104	105 ± 8
NSD1	103	102	107	104 ± 2
NSD2	100	86	90	92 ± 7
NSD3	112	104	115	110 ± 5

bioRxiv preprint doi: https://doi.org/10.1101/2023.10.03.560722; this version posted November 13, 2023. The copyright holder for this preprint (which was not certified by peer review) is the author/funder, who has granted bioRxiv a license to display the preprint in perpetuity. It is made available under aCC-BY 4.0 International license.

TABLE S4. Statistics for data collection and processing, structure solution and refinement of the crystal structure of the nsp14 MTase-TELSAM/SS148 complex. Numbers in parentheses refer to the highest resolution shell. R.m.s.d., root-mean-square deviation.

Crystal	nsp14 + SS148
PDB accession code	8BWU
Space group	P 65
Cell dimensions	a, b, c (Å)<br>109.3 109.3 48.7<br>$\alpha, \beta, \gamma (^\circ)$<br>90.0 90.0 120.0
Resolution range (Å)	35.78 - 2.36 (2.44 - 2.36)
No. of unique reflections	13,850 (1,383)
Completeness (%)	99.5 (98.2)
Multiplicity	19.9 (15.9)
Mean $I/\sigma(I)$	7.63 (0.54)
Wilson B factor ($Å^2$)	46.03
R-merge	0.3783 (3.603)
R-meas	0.3881 (3.722)
CC1/2 (%)	99.5 (42.2)
CC* (%)	99.9 (77.0)
R-work (%)	22.96 (36.39)
R-free (%)	26.43 (32.05)
CC-work (%)	94.6 (64.8)
CC-free (%)	90.9 (78.2)
R.m.s.d. bonds (Å)	0.002
R.m.s.d. angles (°)	0.42
Average B factor ($Å^2$)	overall<br>56.74<br>protein<br>57.08<br>ligands<br>41.81<br>solvent<br>45.58
Clashscore	1.42
Ramachandran (%)	favored<br>98.4<br>allowed<br>1.6<br>outliers<br>0.0

bioRxiv preprint doi: https://doi.org/10.1101/2023.10.03.560722; this version posted November 13, 2023. The copyright holder for this preprint (which was not certified by peer review) is the author/funder, who has granted bioRxiv a license to display the preprint in perpetuity. It is made available under aCC-BY 4.0 International license.

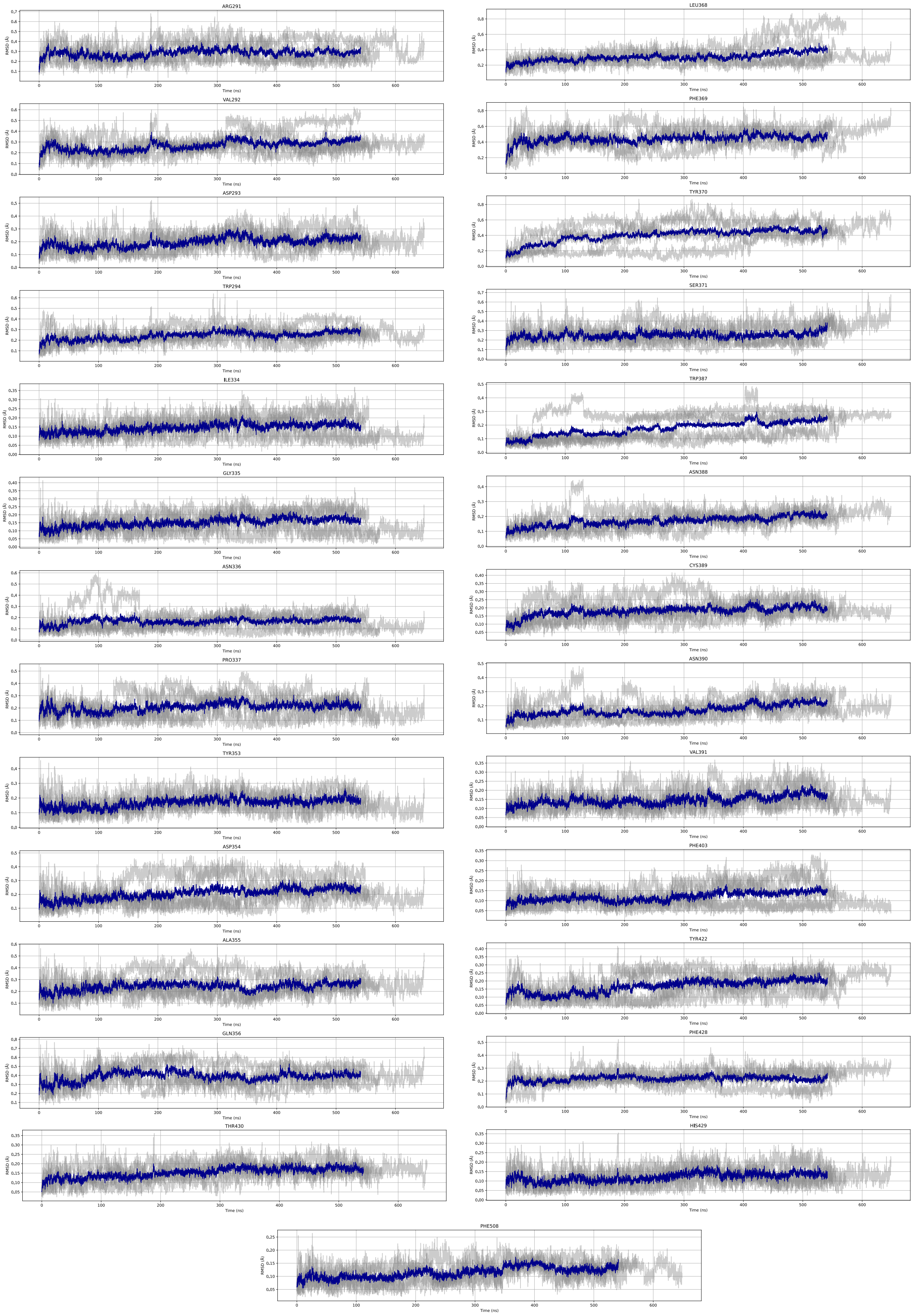

FIG. S7. **RMSD Variation of Residues in the MTase Lateral Binding Pocket Across 300.15K Simulations.** All-atom root mean square deviation (RMSD) movement of residues within the MTase lateral binding pocket during 300.15K simulations across five replicates. The individual RMSD movements for each replicate are depicted as gray lines, representing the diversity of conformational changes across the different simulations. The average RMSD movement, plotted in blue, provides a visual summary of the typical movement exhibited by these residues.
